# Emotional Intelligence (EI) Workshops for Core Trainee Psychiatry Doctors

**DOI:** 10.1192/bjo.2024.309

**Published:** 2024-08-01

**Authors:** Kuljit Mandair, Amitav Narula

**Affiliations:** Black Country Healthcare NHS Foundation Trust, Black Country, United Kingdom

## Abstract

**Aims:**

Emotional Intelligence (EI) is a skill that can help doctors be more effective leaders, work well with others and display the ability of self-control in stressful situations so one is able to act in a calm and rational manner. It is defined as the awareness of one's own emotions and emotions in others and how this affects behaviour. It is a skill that can be developed to allow doctors to manage their emotions to support personal strengths, solve problems and influence the performance of others for positive outcomes.

In the new Core psychiatry curriculum, under 5.1 Team work, trainees are to demonstrate an awareness of how individual personal qualities, emotions and behaviours of both yourself and your team, impact on teamworking and the quality of patient care.

The aim of the workshop was to uncover the definition, science and core components of EI, to reflect on one's own EI and to commit to developing an action plan for building EI skill.

**Methods:**

4 small group-based interactive virtual workshops took place on a monthly basis from September 2022 till December 2022. They were facilitated by a Psychiatry Higher Trainee Emotional Intelligence Practitioner. 28 Black Country Healthcare NHS Foundation trust Core trainees (CT1-CT3) were invited. 60% (17) of trainees attended the 1 hour workshop and completed anonymous feedback at the end of the workshop.

**Results:**

94% of attendees completed anonymous post-workshop feedback. The results showed the following: 100% agreed that the workshop clearly stated and met the objectives, 100% agreed that the workshop covered useful material, 100% felt that it was practical to needs and interests of trainees, 100% felt it was applicable to professional and personal life including mental wellbeing, 100% agreed that the workshop enabled them to reflect on EI skills that can be applied to work, 94% felt that the workshop is relevant and useful to doctors and 100% of participants would recommend this workshop to Psychiatry Doctors and Doctors from other specialities.

**Conclusion:**

It can be concluded that all CT doctors who participated in the EI workshops found them helpful and relevant within their Core Psychiatry Training Programme. All participants found the benefits applicable to both professional and personal life as well as enhancing mental wellbeing. This is reflected by the positive and encouraging anonymous feedback results. Developing awareness of emotions and self-awareness is part of the new Psychiatry curriculum and therefore some teaching/training should be made available to trainee doctors.

